# Effect of a Roughness Element on the Receptivity of a Hypersonic Boundary Layer over a Blunt Cone Due to Pulse Entropy Disturbance with a Single Frequency

**DOI:** 10.3390/e20060404

**Published:** 2018-05-24

**Authors:** Zhenqing Wang, Mingfang Shi, Xiaojun Tang, Hongqing Lv, Lidan Xu

**Affiliations:** 1 College of Aerospace and Civil Engineering, Harbin Engineering University, Harbin 150001, China; 2 Beijing Spacecrafts, China Academy of Space Technology, Beijing 100094, China

**Keywords:** direct numerical simulation (DNS), pulse entropy disturbances, FFT, hypersonic, receptivity

## Abstract

A high-order finite difference method was used to simulate the hypersonic flow field over a blunt cone with different height roughness elements. The unsteady flow field induced by pulse disturbances was analyzed and compared with that under continuous disturbances. The temporal and spatial evolution characteristics of disturbances in the boundary layer were investigated and the propagation of different disturbance modes in the boundary layer was researched through the fast Fourier transform (FFT) method. The effect of the roughness element on the receptivity characteristic of the hypersonic boundary layer under pulse entropy disturbances was explored. The results showed that the different mode disturbances near roughness in the boundary layer were enlarged in the upstream half of the roughness element and suppressed in the downstream half. However, the effect of roughness weakened gradually as the disturbance frequency increased in the boundary layer. A phenomenon of mode competition in the downstream region of the roughness element exited. As the disturbances propagated downstream, the fundamental mode gradually became the dominant mode. A certain promotion effect on the mode competition was induced by the roughness element and the effect was enhanced with the increase in the roughness element height.

## 1. Introduction

The receptivity of the hypersonic boundary layer is the response of the boundary layer to external disturbances. That means the receptivity is the process of excitation and evolution of disturbances in the hypersonic boundary layer. This process defines the initial state of the disturbances in the boundary layer. After the receptivity stage, disturbances in the boundary layer grow linearly or nonlinearly. Therefore, there is an essential effect on the transition of the hypersonic boundary layer.

The receptivity characteristic is the basis for the analysis of the boundary layer stability that directly affects aerodynamic force and aerodynamic heating of a hypersonic vehicle. Compared with low velocity or incompressible flow, the interaction between the hypersonic boundary layer and the shocks or entropy layer makes the flow in the hypersonic boundary layer more complicated. That is why the mechanism of hypersonic boundary layer receptivity is still not fully understood.

**Figure 1:**
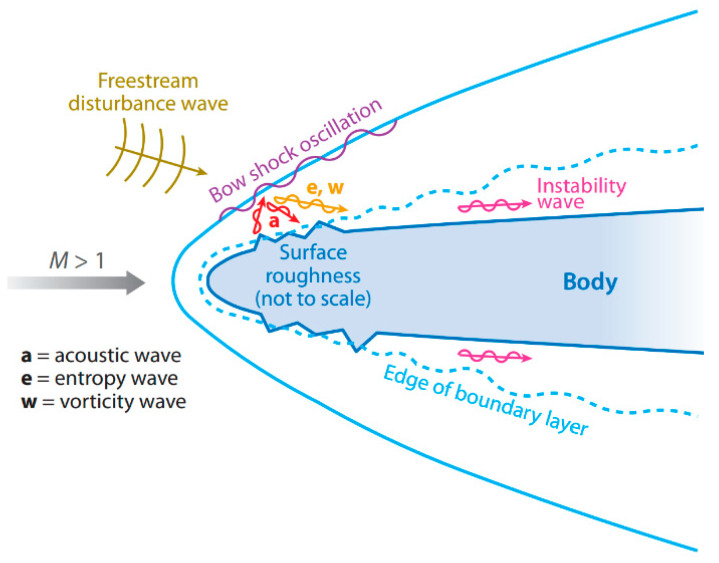
A schematic of flow field under free stream disturbances and surface disturbances [5].

There are many factors that affect the receptivity of the hypersonic boundary layer such as free stream disturbances, wall temperature, and roughness element. Nose-bluntness [1,2,3] and entropy-layer instability [2,3,4] also affect the flow state in the boundary layer. The factors are simply divided into the free stream disturbances and surface disturbances [5], as shown in Figure 1. The type of disturbance has a great influence on the receptivity of the hypersonic boundary layer. Many scholars have analyzed the effect of the types of disturbance on the receptivity of the hypersonic boundary layer through experiment or simulation methods [6,7,8,9,10].

Free stream disturbances include acoustic, vortical, and entropy disturbances while acoustic disturbance can be divided into slow acoustic disturbance and fast acoustic disturbance. Cerminara et al. [11] studied the leading-edge receptivity for supersonic/hypersonic flows over a blunt wedge under acoustic disturbances. Chou et al. [12] analyzed the instabilities generated by free stream laser perturbations developed in the hypersonic boundary layer by experiments in a quiet tunnel. Qin et al. [13] studied the receptivity of the hypersonic boundary layer over a wedge to free stream disturbances including acoustic, vortical, and entropy disturbances. Zhong et al. [14,15,16,17,18] carried out a series of studies to analyze the receptivity of the hypersonic boundary layer over a flat plate and a parabola by direct numerical simulation. Shi et al. [19] and Tang et al. [20] discussed the receptivity under free stream disturbances with different frequency or amplitude and investigated the evolution of disturbances in the boundary layer using the fast Fourier transform (FFT) method. The results presented in previous papers have shown there are still some differences in receptivity mechanisms at different conditions of the same type of disturbances such as single-frequency disturbances and multi-frequency disturbances, small disturbances and finite amplitude disturbances. The former difference can be clarified by the number of disturbance frequency, while the latter difference can be clarified by the amplitude. Classically, small disturbances mean the disturbance amplitude is between 10^−5^ and 10^−4^, while a disturbance amplitude of finite amplitude disturbances is greater, in the area of 10^−2^. It is worth noting that the time length of the free stream disturbances, namely pulse disturbances and continuous disturbances, has a great influence on the receptivity process of the hypersonic boundary layer. Tang et al. [21] pointed out that the amplitude and frequency band of the disturbances in the boundary layer were both impacted by the time length of the free stream disturbances.

Surface disturbances also affect the receptivity characteristic. It is known that surface disturbances can significantly change flow structure and may cause flow separation in the hypersonic boundary layer. The effect of roughness element shape (cylinder, square, and micro-ramp) on the stability of the boundary layer was investigated through experimental method by Duan et al. [22] and numerical simulations by Ye et al. [23]. Zhou et al. [24] also analyzed flat-plate boundary layer stability induced by different shape roughness elements in both the supersonic and hypersonic regimes. Fong et al. [25,26,27] and Wang et al. [28] conducted a parametric study of two-dimensional roughness elements on the stability of the hypersonic boundary layer by a new high-order difference method named the high-order cut-cell method, and the effect of interaction between roughness elements and free stream disturbances on the receptivity of the hypersonic boundary was also investigated. Previous investigations on receptivity characteristics under different parameters of roughness elements including location, height, shape, and so on, have shown that roughness elements have an essential impact on the receptivity mechanism. The influence of interaction between the roughness elements and free stream disturbances on receptivity is very complicated. In fact, most of these investigations mainly analyzed the effect of interaction between the roughness elements and free stream acoustic disturbances with multi-frequency and attained the evolution process of disturbances in the boundary layer. However, there have been few investigations that have studied the receptivity of the hypersonic boundary layer over a blunt cone with roughness element under free stream disturbances with a single frequency. It is reasonable to believe that there are differences of receptivity mechanism under the two conditions of free stream disturbances.

The focus of this paper is on the effect of the roughness elements on the receptivity of the hypersonic boundary layer over a blunt cone under finite amplitude entropy disturbances with a single frequency for a short period of time. Steady and unsteady flow fields were simulated numerically by the high-order finite difference method, and the effect of the roughness element height on the hypersonic flow field and boundary layer was analyzed. The temporal and spatial evolution characteristics of disturbances in the boundary layer were analyzed, and the propagation of different disturbance modes in the boundary layer was researched by the fast Fourier transform (FFT) method. The influence of the roughness element height on the evolution of disturbances in the boundary layer was also investigated.

## 2. Governing Equations and Numerical Methods

A high-order finite difference method was used to directly conduct the numerical simulation of a hypersonic flow field over a blunt cone with roughness elements to get the effect of roughness elements on the receptivity of the hypersonic boundary layer. The governing equations for the simulation were the two-dimensional Navier–Stokes (N-S) equations in the conservative form at Cartesian coordinates. For convenience, the N-S equations for compressible flow were transformed into curvilinear coordinates and can be expressed as

(1) $$\frac{\partial U}{\partial t}+\frac{\partial F_{i}}{\partial x_{i}}+\frac{\partial F_{\upsilon i}}{\partial x_{i}}=0\;\left(i=1,2\right)$$

where ***U***, ***F****_i_*, and ***F****_υi_* are the vectors of flow variables, convective flux, and viscous flux in the *i*-th spatial direction, respectively, i.e.,

(2) $$\begin{array}{l} U=\left[\rho ,\rho u_{1},\rho u_{2},\rho e\right],\\ F_{i}=\left[\begin{array}{l} \rho u_{i}\\ \rho u_{1}u_{i}+p\delta_{1}{}_{i}\\ \rho u_{2}u_{i}+p\delta_{2}{}_{i}\\ (\rho e+p)u_{i} \end{array}\right],\\ F_{\upsilon i}=\left[\begin{array}{l} 0\\ \tau_{1}{}_{i}\\ \tau_{2}{}_{i}\\ \tau_{ij}-q_{i} \end{array}\right], \end{array}$$

where the variables *ρ*, *u*, *p*, *δ*, *e*, and *τ* are the density, velocity, pressure, Kronecker symbol, total energy, and shear stress, respectively. Only the perfect gas hypersonic flow was considered and

(3) p=ρRT

(4) $$e=\rho C_{v}T+1/2u_{j}u_{j}$$

(5) $$\tau_{ij}=\mu \left(\frac{\partial u_{i}}{\partial x_{j}}+\frac{\partial u_{j}}{\partial x_{i}}\right)+\delta_{ij}\lambda \frac{\partial u_{k}}{\partial x_{k}}$$

(6) $$q_{i}=-k\frac{\partial T}{\partial x_{i}}$$

where *R* is the gas constant and *C_v_* is the specific heat, which is constant with a given ratio of specific heats γ. The viscosity coefficient *µ* was attained by Sutherland’s law, and *λ* is assumed to be −2/3 *µ.* The heat conductivity coefficient *k* can be computed by the Prantl number.

Since the high-order finite difference method can accurately simulate the hypersonic flow field under complex conditions and accurately capture the tiny flow structure in the boundary layer, it has been widely used for the direct numerical simulation of the hypersonic unsteady flow field [29,30]. The central difference scheme has good robustness and convergence, so it is widely employed in the simulation of the hypersonic flow field. However, the central difference scheme may cause serious numerical oscillations in discontinuities or high adverse pressure gradient regions such as shock regions and the higher-order schemes produce more serious numerical oscillations. Therefore, a weighted essentially non-oscillatory (WENO) scheme that could effectively suppress the oscillatory behavior in the discontinuous regions and accurately capture the shocks in the flow field was introduced to provide adequate accuracy level for DNS [31]. In addition, although upwind schemes do not produce numerical oscillations, large numerical dissipation exits in the discontinuities or high adverse pressure gradient regions. In order to maintain the advantage of the upwind schemes and reduce or eliminate the numerical dissipation, the Steger–Warming (S-W) splitting method was employed to split the convective flux [32]. Therefore, the viscous flux in the governing equations was solved by the sixth-order central difference scheme. Convective flux was split into positive and negative flux terms, and then the flux terms were solved by the fifth-order WENO. At the same time, a third-order total variation diminishing the Runge–Kutta (R-K) scheme was employed for time integration. More details of the validation of the numerical program have been discussed in previous papers and the validation is not given here [19,20,21].

## 3. Calculation Model and Conditions

**Figure 2:**
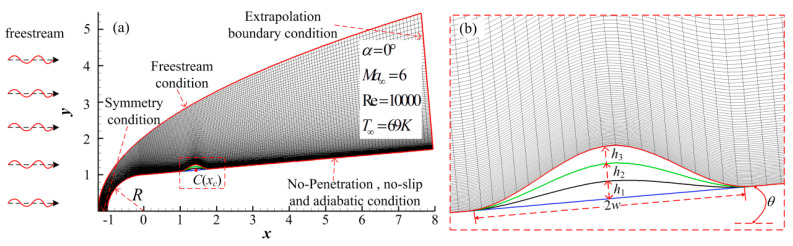
Computational model and mesh grid.

The computational model is a hypersonic flow over a blunt cone with different height roughness elements. The free stream condition and extrapolation condition were employed at the upstream boundary and the outflow, respectively. A symmetry condition was used at *y* = 0, and no-penetration, no-slip, and adiabatic conditions were used on the wall. The computational condition, flow parameters, and mesh grid are shown in Figure 2. *α*, *Ma*, and *Re* denote the attack angle, Mach number, and Reynolds number, respectively. The variables with subscript “∞” denote free stream parameters. A 300 × 120 grid was used to solve the governing equations, and the grid mesh was clustered in a wall-normal direction near the wall and blunt nose, where a bow shock exits. The mesh grid density, which introduces the present computations, matched that in the investigations with a similar computational model conducted by Duan et al. [33], Zhang et al. [34], and Prakash et al. [35], and the grid sensitivity analysis has also been discussed previously. The results showed that the grid was reliable.

The roughness elements shown in Figure 2b were controlled by a third-order polynomial, written as

(7) $$y=\frac{R_{n}+h}{\cos\theta }+x\tan\theta -\frac{3h}{\cos\theta }{\left(\frac{x-x_{c}+h\sin\theta }{h\sin\theta \pm w\cos\theta }\right)}^{2}+\frac{2h}{\cos\theta }{\left(\frac{x-x_{c}+h\sin\theta }{h\sin\theta \pm w\cos\theta }\right)}^{3}$$

where the radius of nose *R_n_* and half cone angle *θ* were 1 mm and 5°, respectively. The parameters of roughness element *h*, *w*, and *x_c_* were the height, half weight, and midpoint coordinate, respectively. The thickness *δ_bl_* of the boundary layer under flow conditions in this paper at the midpoint of the roughness element was 0.1992, so the heights of the roughness element were 0.05, 0.10, and 0.15 which were equal to 25%*δ_bl_*, 50%*δ_bl_,* and 75%*δ_bl_*, respectively. The shapes of the roughness element are summarized in Table 1.

The steady flow field was computed first, and the pulse entropy disturbances, which are accompanied by the free stream entering into the flow field, were then impinged on the upstream boundary. Pulse entropy disturbances can be expressed as Equation (8). The superscripts “ ′ ” used in the following denote perturbation values, which were obtained by the variables’ value of instantaneous flow minus the variables’ value of the local steady flow. The variables *ε*, *k,* and *f* were the amplitude, wave number, generalized frequency of disturbances, respectively, and *ε* = 6 × 10^−2^, *k* = 3.1446 × 10 ^−4^, and *f* = 50π. Therefore, the period of disturbances was 4. The amplitude and period of disturbances characterized their intensity and time, respectively. The focus of this paper was to reveal the influence of pulse entropy disturbances on the hypersonic boundary layer, so the disturbances only existed for half a period from *t* = 0 to *t* = 2. The incidence angle of free stream disturbances was 0.

(8) $$\left[\begin{array}{l} u^{\prime }\\ v^{\prime }\\ p^{\prime }\\ \rho^{\prime } \end{array}\right]=\left[\begin{array}{l} 0\\ 0\\ 0\\ \varepsilon Ma \end{array}\right]e^{i(kx-\frac{fRe}{10^{6}}t+\frac{\pi }{2})}.$$

The variables used in the following were all dimensionless and *R_n_*/*u_∞_*_,_ *ρ_∞_*, *p_∞_*, *u_∞_*/*Rn*, *Rn*, *T_∞_*, *μ*_∞_, *k*_∞_, and *u*_∞_ were used for non-dimensionalizing time *t*, density *ρ*, pressure *p*, frequency *f*, length (including *x*, *y*, *h*, and *w*), temperature *T*, viscosity coefficient *µ*, heat conductivity coefficient *k*, and velocity, respectively.

## 4. Results

### 4.1. Effect of the Roughness Element on the Hypersonic Flow Field

**Figure 3:**
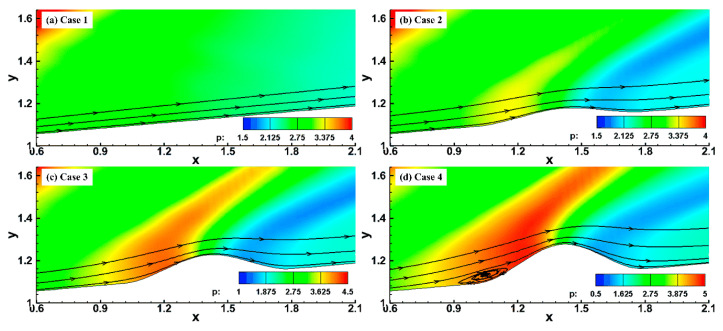
The pressure distribution near roughness elements.

**Figure 4:**
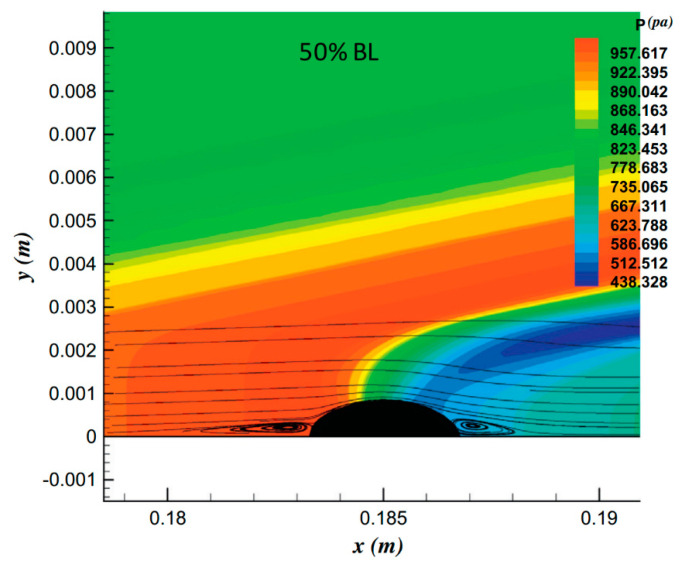
The pressure distribution near the roughness element of the flat plate [27].

To analyze the effect of pulse entropy disturbance on a hypersonic flow field over a blunt cone, the steady flow field was obtained using the high-order finite difference method. Figure 3 shows the pressure and streamline patterns of steady hypersonic flow field near the roughness elements in the near-wall region. It can be seen that the roughness elements had a great influence on the hypersonic flow field and boundary layer. The flow was compressed in the upstream and downstream region of the roughness element, and the degree of compression increased with increasing roughness element height. Even flow separation exited near the leading edge of the roughness element when the height of the roughness element increased to a certain extent, as shown in Figure 3d. The compression effect of the roughness element resulted in the formation of two compression waves in the flow field, and the roots of the compression waves were located in the leading and the trailing edge regions of the roughness element, respectively. A bundle of expansion waves formed above the roughness element. This was similar to the results obtained by Fong [27] when analyzing the effect of the roughness elements on the stability of the hypersonic boundary layers on flat plate, and the steady flow field is shown in Figure 4. Fong [27] also pointed out that the roughness element led to the formation of two compression waves that were parallel to the bow shock in the vicinity region of the upstream and downstream of the roughness element, but the location and length of the vortex were significantly different from the results in this paper. Park [36] and Ye [23] also found that flow separation occurred at the leading edge of the roughness element by numerical simulation and experimental methods, which was consistent with the results in this paper. The effect of the roughness element on the flow separation in the hypersonic boundary layer is very complicated. Generally speaking, flow separation is mainly caused by adverse pressure gradient. For the flow near the roughness element, the flow in the boundary layer undergoes an expansion acceleration process in the middle of the roughness element that makes the flow separation in the downstream region of the roughness element more difficult. The adverse pressure gradient in the downstream region of roughness elements for these cases is not enough for flow separation.

**Figure 5:**
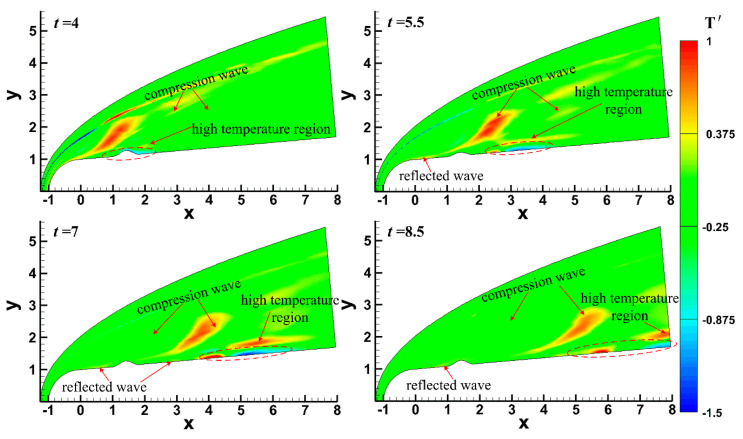
Temperature perturbation contour at different times under Case 4.

**Figure 6:**
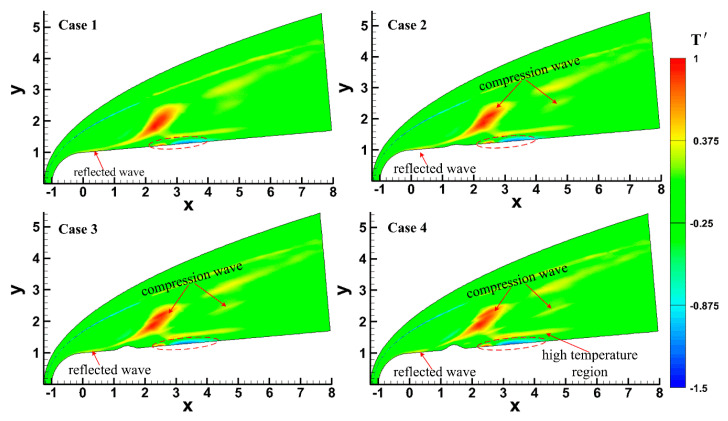
Temperature perturbation contour under different roughness element heights at *t* = 5.5.

Pulse entropy disturbances with single-frequency were introduced into the steady flow field to analyze the effect of the roughness element on an unsteady hypersonic flow field over a blunt cone, and the effect of the roughness element on the evolution process of disturbances in the boundary layer is discussed emphatically. Figure 5 shows the distribution of temperature perturbation at different times under the condition of Case 4, and Figure 6 shows the distribution of temperature perturbation under different roughness element heights at *t* = 5.5. It can be seen that a diagonal-strip area where the temperature perturbation was large formed in the flow field when entropy disturbances entered into the flow field. It has been pointed out above that two compression waves formed in the flow field, and a deformation of compression waves formed when entropy disturbances passed through the compression waves. The temperature perturbation had a certain degree of increase in the deformation region, and the degree of increase increased with the roughness element height, as shown in Figure 6.

As for the boundary layer, the temperature perturbation increased and then decreased after entropy disturbance entered the boundary layer, as shown in the dotted circle in Figure 5 and Figure 6. As the disturbances in the boundary layer evolved downstream (*t* = 4~7), disturbances grew rapidly and then slightly decayed (*t* = 7~8.5). A high-temperature perturbation zone was generated at the edge of the boundary layer and temperature perturbation increased as entropy disturbances propagated downstream. It should be noted that the effect of disturbances did not disappear when the disturbance flowed away. Under the effect of reflected waves in the boundary layer, the temperature perturbations still had a certain degree of increase.

**Figure 7:**
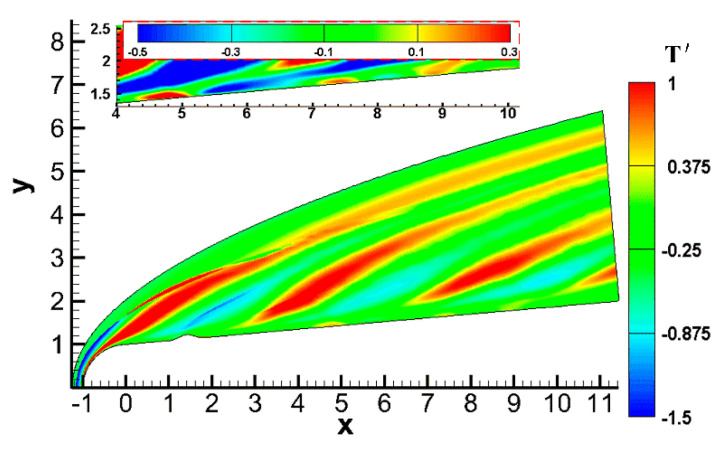
Temperature perturbation contour under continuous waves.

The unsteady flow field under pulse disturbances is essentially different from that under continuous disturbances. Figure 7 shows the temperature perturbation in an instantaneous hypersonic flow field under continuous slow acoustic disturbances, which are the same as the model in Case 2 (the parameters of free stream and disturbance were the same as those in this paper, and *x*_c_ = 1.4071, *w* = 0.40, and *h* = 0.10). In order to more clearly see the influence of the boundary layer under continuous slow acoustic disturbances, the distribution of temperature perturbation was enlarged in the upper left corner of Figure 7. It can be seen that the effect of continuous slow acoustic wave disturbances on the flow field was significantly different from that of pulse entropy disturbances: (1) the continuous slow acoustic wave disturbances continued to affect the hypersonic flow field, resulting in the deformation of bow shock, the intermittent formation of an oblique strip-shaped high-temperature region, and a wider influence range in the flow field; (2) compared with the pulse entropy disturbances, the variation range of temperature perturbation in the boundary layer under continuous slow acoustic disturbances was small. It can be seen that several high-temperature zones and low-temperature zones were alternately formed in the boundary layer.

Again, it has been proven that there are essential differences between pulse wave disturbances and continuous wave disturbances, slow acoustic disturbances, and entropy disturbances on the hypersonic flow field and boundary layer over a blunt cone with roughness elements, which also explains the necessity of this study.

### 4.2. Effect of the Roughness Element on the Evolution of Disturbances in the Hypersonic Boundary Layer

**Figure 8:**
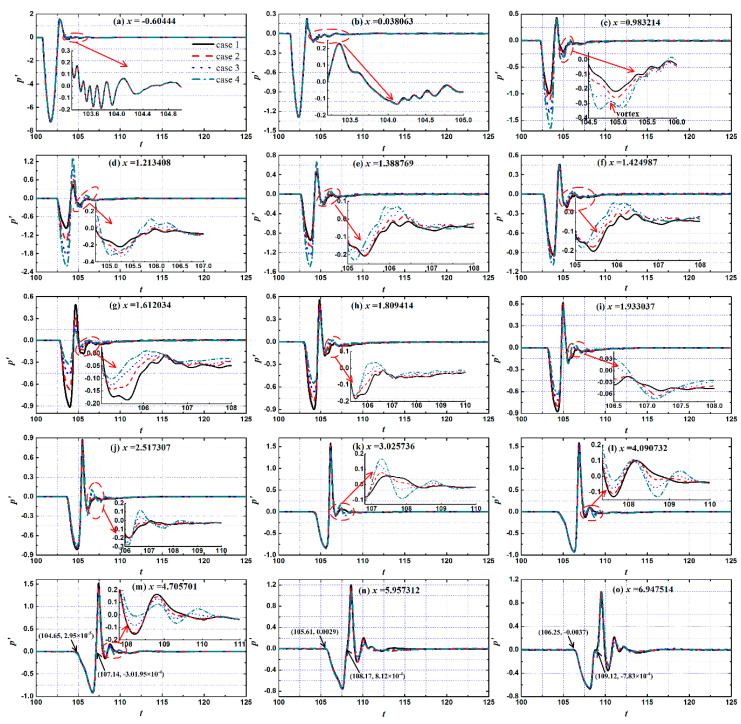
Time history trace of wall pressure perturbation at various streamwise locations.

The thermodynamic state in the boundary layer under pulse entropy disturbances was significantly changed. Hirschel [37] indicated that the change in thermodynamic state had a significant impact on the flow structure and state in the boundary layer, so the flow state in the boundary layer changed under pulse entropy disturbances. Figure 8 shows the evolution of pressure disturbances in the time domain at different locations in the boundary layer on different height roughness elements under pulse entropy disturbances. Several stations along the flow direction were selected for analyzing the evolution process of disturbances in the hypersonic boundary layer: (1) the upstream stations of the isolated roughness element (*x* = −0.60444~0.038063, 2 stations); (2) the stations around the roughness element (*x* = 0.983214~1.809414, 6 stations); (3) the downstream region of the roughness element (*x* = 1.933037~6.947514, 7 stations). It can be seen that the evolution process of the disturbances in the boundary layer was similar at different positions. As the disturbances passed through a certain position, the wall pressure first decreased, increased, and then oscillated and decayed to the undisturbed state.

Here, the time of the pulse entropy disturbances was very short, only half a period, while the response time of the boundary layer was more than half a period. Therefore, the evolution time of disturbances in boundary layer was divided into two stages: (1) the disturbance phase—the half period time after the disturbance wave was felt from this location (∆*t* = 2)—and (2) the dissipation phase—the phase that presents a damped oscillation attenuation process of disturbances after the disturbance phase until the boundary layer recovered to the undisturbed state. In the dissipation phase, reflected waves reflect continuously between the wall and shock after passing through the shock, also shown in Figure 6. After the reflected wave passed through a certain position on the wall, the reflected wave did not disappear and continued to propagate at this position with damping oscillation. The influence of the reflected wave in the boundary layer was mainly concentrated in the dissipation phase.

The roughness element had a great influence on the evolution of the disturbance in the spatial domain in the vicinity and downstream region of the roughness element, as shown in Figure 8a,b, while there was no influence on the upstream region. In the disturbance phase, the influence of the roughness element on the evolution of disturbances in the boundary layer was mainly reflected in the variation in amplitude. The disturbances were amplified significantly in the upstream half region of the roughness element, and the amplification effect increased significantly with the increase in the height of roughness element. At *x* = 1.213408, the amplitude of pressure perturbation was twice as large in Case 4 as in Case 1. The amplitude of pressure perturbation was significantly suppressed in the downstream half region of the roughness element. At *x* = 1.612034, the pressure perturbation amplitude in Case 4 was even less than half of that in Case 1, and this suppression continued for a distance along the flow direction. 

Fong and Tang [38,39] drew similar conclusions when they analyzed the effect of two-dimensional roughness element on the stability of the boundary layer on the flat plate at Mach 6. The roughness element resulted in an increase in the pressure in the upstream half region of the roughness element and a decrease in the downstream half region. This effect was limited in the region near the roughness element. As disturbances evolved to the downstream, the period length of the disturbance phase increased gradually, as shown in Figure 8m–o, which indicate that the dominant mode of the disturbances migrated to low frequency.

The effect of the roughness elements of disturbances in the dissipation phase was very different from that in the disturbance phase. In the downstream region of the roughness element (x > 1.424987), the waveform of the disturbances in the boundary layer under the roughness element was closer to the sinusoidal mode waveform, which is the waveform of the free stream disturbances. This showed that the roughness element had a filtering effect on the disturbances in the boundary layer, and some disturbances with small amplitude were filtered by the roughness element in the boundary layer. In addition, it should be pointed out that the vortex formed at the leading edge of the roughness element at x = 0.983214 in Case 4 had some influence on the evolution of disturbances in the boundary layer, as shown in Figure 8c.

It has been pointed out above that the dominant mode transformation phenomenon occurred in the propagation of disturbances to downstream in the boundary layer. In order to reveal the effect of roughness element on the evolution mechanism of different mode disturbances in the hypersonic boundary layer, the fast Fourier transform (FFT) method was used to transform the time domain signal into the frequency domain. Furthermore, the influence of roughness element height on the evolution process of disturbances was also researched. The FFT formula is expressed as follows:

(9) $$p^{\prime }(x,y,t)=Re(\sum_{n=0}^{N}\left|{p^{\prime }}_{n}(x,y)\right|e^{i\left[-n\omega t+\varphi n(x,y)\right]}).$$

**Figure 9:**
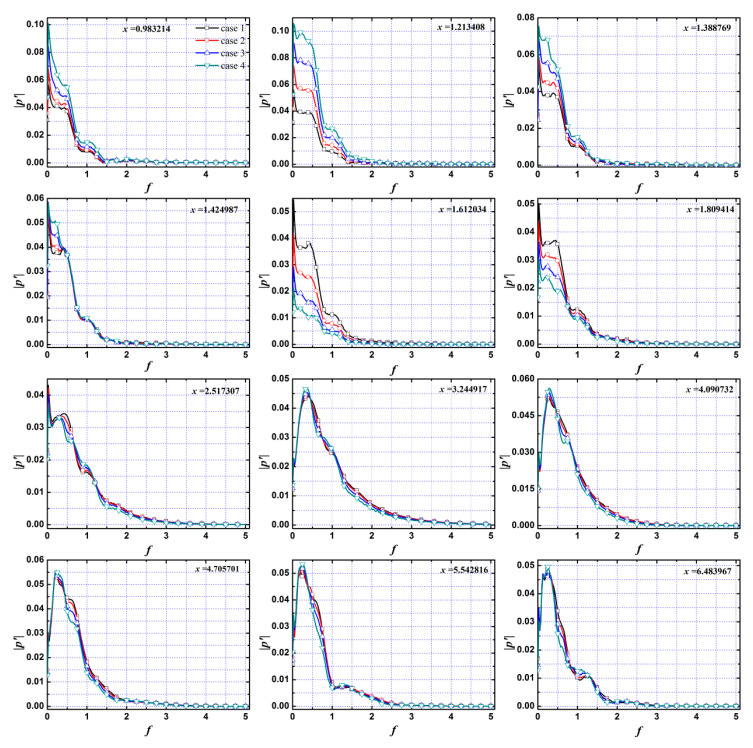
The spectrum of disturbances at different locations in the boundary layer.

Figure 9 is the spectrum diagram of pressure perturbation in the boundary layer with different roughness element heights under pulse entropy disturbances. Since the roughness elements do not affect the evolution of the disturbances in the nose region of a blunt cone (*x* < 0), the evolution process of the disturbances in this region is not given. In general, the amplitude of the disturbances decreased gradually in the process of propagation to the downstream, and the amplitude of the disturbances was increased by the roughness element in the first half region of the roughness element and decreased in the half region downstream in the boundary layer. The variation in the amplitude of pressure perturbation caused by the roughness element increased with the increase in roughness element height. The frequency band of the disturbances gradually widened in the process of propagation of the disturbances to the downstream, and the band was widest at *x* = 3.244917, then gradually narrowed. However, the dominant mode gradually decreased in that propagation process, and the proportion of low frequency mode (*f* < 0.5) gradually increased. Fong et al. [25] also found that the dominant mode in the downstream region of the roughness element migrated to low frequency when investigating the effect of the roughness element on the evolution of the disturbances in the flat plate boundary layer under free stream pulse disturbances.

**Figure 10:**
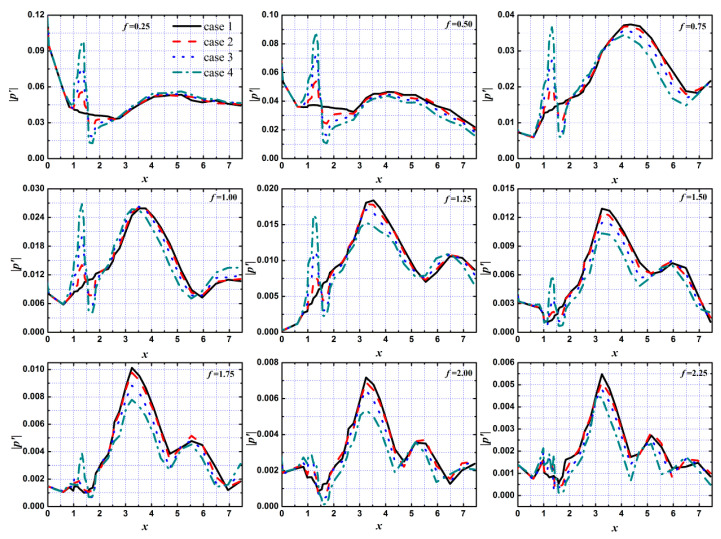
Evolution of different frequency of disturbance wave along streamwise with different roughness element heights.

In order to more clearly show the evolution process of different mode disturbances streamwise under different roughness element heights, Figure 10 shows the evolution of the disturbances at the fundamental and harmonic modes (fundamental mode *f* = 0.25, harmonic modes *f_n_* = 0.25n, *n* = 1, 2, 3, ...) in the boundary layer. It can be seen that the roughness elements had a great influence on the evolution of different mode disturbances, and the influence was mainly concentrated in the roughness elements and their downstream region. 

For the near roughness element region, the disturbances with different modes were amplified in the first half region of the roughness element and suppressed in the second half region, and the amplification/suppression effect was enhanced with the increase in roughness element height. However, the effect gradually weakened with the increase in the frequency of disturbances. When the frequency of disturbance was *f* > 1.75, the influence of the roughness element height on the evolution of disturbances in the region near the roughness element was very small.

For the downstream region of the roughness element, the propagation of disturbances with different modes showed different trends: some modes, such as the fundamental mode and the second-order harmonic mode, were suppressed after a slight increase in the downstream region of the roughness element, and the attenuation of the fundamental mode was smaller than that of the second-order harmonic mode; some modes, such as third-order harmonic mode, decayed rapidly after the rapid growth in the downstream region of the roughness element; some modes, such as the fifth-order harmonic mode, underwent the process of growth and attenuation. The disturbances in the boundary layer tended to decay in the process of propagation to the downstream region, while the change in fundamental mode was relatively gentle. The harmonic modes decayed or even rapidly decayed after growth, which indicated that there was a physical phenomenon of mode competition in the process of propagation of the disturbances to the downstream in the boundary layer and the dominant mode had a certain inhibition effect on the other modes. It is well known that disturbances with larger amplitude are accompanied by larger disturbance energy, which indicates that the mode competition phenomenon of the disturbances in the boundary layer was accompanied by energy migration. 

The roughness element height had a certain suppression effect on the propagation of the disturbances with different modes except for the fundamental mode in the downstream region, and the distance of the suppression effect induced by the roughness element on the evolution of the different modes was very different: some modes, such as the second-order harmonic mode, were sustainably suppressed in the downstream region of the roughness element; some modes, such as the fundamental mode, were amplified after being restrained by the roughness element; some modes, such as the third-order harmonic mode, underwent the process of growth attenuation several times in the downstream region of the roughness element. That is to say, the growth of the fundamental mode was promoted, while the harmonic modes were inhibited by the roughness in the downstream region of the roughness element in the boundary layer, so the increased roughness element height could promote the phenomenon of mode competition in the hypersonic boundary layer.

## 5. Conclusions

A hypersonic steady and unsteady flow field over a blunt cone with different height roughness elements under free stream with and without pulse entropy disturbances was simulated by direct numerical simulation (DNS). The influence of the roughness element height on hypersonic flow field and boundary layer was investigated. The evolution process of disturbances in the boundary layer was revealed by the fast Fourier transform (FFT) method, which was used to transform the time domain signal into the frequency domain. The results showed the following:

(1) A diagonal-strip high-temperature area was formed after the pulse entropy disturbances entered the hypersonic flow field and was distorted by the interaction with compression waves caused by the roughness element. A local high temperature region was formed in the deformed area. With the increase in roughness element height, the interaction between pulse entropy disturbances and compressional wave was enhanced.

(2) The amplitude of disturbances in the boundary layer gradually decreased in the process of disturbances propagating to the downstream. However, the amplitude of disturbances was amplified by the roughness element in the upstream half region of the roughness element and was inhibited in the downstream half region. The effect of the roughness element was enhanced with the height increase. Furthermore, the roughness elements had a filtering effect on the reflected waves in the dissipation phase of the evolution progress of disturbances in the boundary layer.

(3) The amplitude of different modes increased in the upstream half region of the roughness element and decreased in the downstream half region, and the variation in the amplitude of disturbances increased with the increase in the roughness element height. However, disturbances with higher frequency in the boundary layer were not sensitive to the roughness elements. There was little influence of the roughness element on the disturbances with frequency *f* > 1.5 in the boundary layer. The phenomenon of mode competition where harmonic modes were suppressed and the fundamental mode was promoted by the roughness element exited in the propagation process of the different modes to the downstream region of the roughness element. Roughness element could promote the mode competition phenomenon.

## Figures and Tables

**Table 1:** Shapes of roughness elements.

	*x*_c_	*w*	*h*
Case 1	1.4071	0.40	0
Case 2	1.4071	0.40	*h*_1_ = 0.05 (25%*δ_bl_*)
Case 3	1.4071	0.40	*h*_2_ = 0.10 (50%*δ_bl_*)
Case 4	1.4071	0.40	*h*_3_ = 0.15 (75%*δ_bl_*)
